# Climate change adaptation: Where does global health fit in the agenda?

**DOI:** 10.1186/1744-8603-8-10

**Published:** 2012-05-27

**Authors:** Kathryn J Bowen, Sharon Friel

**Affiliations:** 1 National Centre for Epidemiology and Population Health, Australian National University Department of Resource Management and Geography, University of Melbourne, Carlton, Victoria 3010, Australia, Canberra, ACT 0200, Australia

**Keywords:** Global health, Climate change, Adaptation, Equity, Sustainable development, Adaptation funding, Social determinants

## Abstract

Human-induced climate change will affect the lives of most populations in the next decade and beyond. It will have greatest, and generally earliest, impact on the poorest and most disadvantaged populations on the planet. Changes in climatic conditions and increases in weather variability affect human wellbeing, safety, health and survival in many ways. Some impacts are direct-acting and immediate, such as impaired food yields and storm surges. Other health effects are less immediate and typically occur *via* more complex causal pathways that involve a range of underlying social conditions and sectors such as water and sanitation, agriculture and urban planning. Climate change adaptation is receiving much attention given the inevitability of climate change and its effects, particularly in developing contexts, where the effects of climate change will be experienced most strongly and the response mechanisms are weakest. Financial support towards adaptation activities from various actors including the World Bank, the European Union and the United Nations is increasing substantially. With this new global impetus and funding for adaptation action come challenges such as the importance of developing adaptation activities on a sound understanding of baseline community needs and vulnerabilities, and how these may alter with changes in climate. The global health community is paying heed to the strengthening focus on adaptation, albeit in a slow and unstructured manner. The aim of this paper is to provide an overview of adaptation and its relevance to global health, and highlight the opportunities to improve health and reduce health inequities *via* the new and additional funding that is available for climate change adaptation activities.

## Introduction

Adapting to climate change is now seen as a core component of our climate change response arsenal. This is because, unfortunately, climate change mitigation strategies alone will not prevent adverse events resulting from existing climate change; we are now too far down the climate change road to avoid the repercussions of more severe weather events, changes to agricultural yields, conflict and displacement, and the health effects that arise from all of these impacts. Although adaptation has received much attention, both practically and in climate change research in the last decade or so, climate change adaptation for health has only recently begun to emerge in the public health literature and policy discourse. The objectives of this paper are to: i) outline the relevance of climate change adaptation to global health, ii) highlight the importance of linking the social determinants of health and sustainable development agendas with climate change adaptation measures, iii) provide an overview of the global health and climate change adaptation activities thus far, and present some examples of activities relevant to health, and iv) describe the (constantly evolving) main adaptation financial mechanisms for developing countries and implications of these for global health. The paper concludes by arguing that the global health community can use the opportunities provided by the increasing flow of funding to climate adaptation to address existing and future health burdens.

## Adaptation – an overview and its relevance to global health

Adapting to environmental variability has been a focus of anthropological study since the early 1900s [1]. The term adaptation was first applied to the study of the consequences of human-induced climatic change in the 1990s. Adaptation is generally understood to mean an adjustment in social-ecological systems in response to environmental changes and their impacts. A central goal of adaptation is the development of adaptive capacity, without which, adaptation is less likely to occur. Adaptation strategies must be flexible and able to incorporate new hazard information, as well as information on socio-economic and environmental systems [2]. Theoretically, adaptation activities should be developed based on a sound assessment of a particular population’s vulnerability (a ‘vulnerability assessment’ or VA) – *i.e.* who is most vulnerable, what are they most vulnerable to, and to what extent do they have the capacity to cope or adapt - but the actual conduct of a rigorous VA to inform adaptation planning and programs is not commonplace and often inadequate. Vulnerability assessments and resultant adaptation plans have been carried out at various scales and sectors by research institutions, non-governmental organisations, United Nations agencies and national and sub-national governments. For least-developed countries, the process of completing a National Adaptation Programme of Action (NAPA), which combines a vulnerability assessment and the development of adaptation activities, is a precursor to receiving adaptation financing through the United Nations Framework Convention on Climate Change (UNFCCC).

Different types of adaptation exist and have been commonly defined as i) anticipatory and reactive, which indicates adaptation activities that either take place before the impacts of climate change or in response to a climate change impact, ii) public and private investment and iii) autonomous and planned, which contrasts deliberate public policy decisions with those that occur naturally by private actors [3]. Uncertainty in future climate conditions presents a challenge for decision-making and the development of adaptation (and mitigation) activities [4] (see Adger *et al.*, 2007 for review). Various sectors, including water infrastructure, land-use planning, building and housing and transport infrastructure are identified as sectors that despite this uncertainty should already be taking climate change into account in developing adaptation strategies [5], but the health sector is often missing in such analyses, despite health being closely linked to various sectors (Table 1). As shown in Table 1, the health sector is responsible for a variety of adaptation activities, ranging from disease surveillance to managing child malnutrition. However, this table also presents the health sector as an important partner for many other activities that have links to health, but which the health sector may not have direct responsibility for, such as supporting food security and responding to disasters.

**Table 1:** Examples of health-related adaptation activities and relevant sector involvement

	**Responsible sector**	**Partner sectors**
**Adaptation activity**		
Improved and increased surveillance for infectious diseases (particularly diarrhoeal disease, malaria and dengue)	Health	Water Supply/Sanitation/Infrastructure
Developing community-based models of management of children with acute malnutrition	Health	Agriculture/Community services
Develop sustainable methodologies for improving rice production practices	Agriculture	Health/Agriculture/Rural Development
Improve and strengthen the capacity of disaster management authorities to effectively coordinate and plan emergency response to droughts, floods and storms	Disaster Management	Health/Finance
Provide water filters for household use	Rural Development	Water Supply/Sanitation/Infrastructure/Health/Finance

## Climate change, social determinants of health and sustainable development

Changes in climatic conditions and increases in weather variability affect human well being, safety, health and survival in many ways [6]. Although some vector-borne diseases will expand their range and seasonality, and death tolls will increase because of heatwaves, the indirect effects of climate change on basic human needs such as food, water and shelter will be likely to have the biggest effect on global health [7]. A substantially increased fraction of the world's population is likely to face severe food shortages and water insecurity by the end of the century due to climate change. Climate change is likely to impair crops, herds and fishstocks through rising temperatures, water stress, extreme weather events, spreading plant and animal diseases, sea level rise and ocean acidification. The health of millions of people will be compromised through an increase in the frequency of intense hurricanes, cyclones, and storm surges causing flooding and direct injury, increasing the health risk among those living in urban slums and where shelter and human settlements are poor [8]. With this will come unemployment, homelessness, dislocation, migration and conflict. All of these may substantially increase levels of stress, anxiety and depression, impairing mental as well as physical health.

Much of the emerging global variation in health impacts of climate change is arguably due to existing economic, social and health inequities [9]. Key elements of our modern global world – the asymmetric distribution of power, income, goods, and services shaped by political, economic and social forces and norms and value in society, and the consequent unfairness in the immediate conditions of daily living (access to health care, access to nutritious foods, conditions of work and shelter, and the nature of communities and cities) have widened health inequities [10]. It is likely that inequities in these ‘social determinants’ of health also create inequities in climate change adaptive capacity. This is seen in the differing national and sub-national climate change vulnerabilities such as existing levels of heat and food stress, and exposure to disease vectors, and differing capacities to adapt to changing climatic conditions.

Take urbanisation for example. Urban living conditions already affect the health of more than half of the world’s population. And urban living can amplify the health effects of climate change [11,12]. Sea-level rise has profound implications for the 13% of the world’s urban population living in the low elevation coastal zone [13]. Low-lying cities and towns near coasts face increased risks from more frequent and more intense hurricanes, cyclones, and storm surges causing flooding, direct injury and damage to infrastructure, including roads, housing, water and sanitation systems. Poorer urban households are usually at higher direct health risk due to weaker structures, less safe city locations and building sites, and the weaker resilience of infrastructure in poorer cities to withstand damage There may also be other health consequences [7]. Poorer urban households also often lack the economic resources to evacuate in the face of climate-related disasters, or to rebuild damaged structures. And they typically lack the political influence to secure protective policies [14].

Many cities in low and middle income countries are built in marginal lowlands, which are particularly hot, humid, and often plagued by disease, thereby exposing people who live there to a variety of heat-related health risks [15]. Poor neighbourhoods with weak infrastructure, buildings and unplanned settlement developments with little green spaces, and use of black corrugated steel panels for roofs and walls are likely to be more exposed to high temperatures compared to more affluent neighbourhoods, and will have less capacity to adapt to the impact [16]. Lower socio-economic groups are more likely to be those urban workers exposed to working conditions with excess heat and therefore at increased risk compared to higher social status groups [17].

Poor living conditions, particularly those among the one billion people living in urban slums are the breeding ground for climate-sensitive infectious diseases such as diarrhoea, malaria and dengue [18]. When basic infrastructure is inadequate, existing conditions of poor sanitation and drainage and impure drinking water are further stressed under conditions of extreme weather events and flooding, leading to the transmission of infectious diseases, which puts poor urban households at higher than usual risk. Around half of the urban population of Africa and Asia lacks provision for water and sanitation to a standard that is healthy and convenient. In Latin America and the Caribbean, more than a quarter lack such provision.

There is a significant risk that declining agricultural productivity growth, growing competition for land and water, and increasing food demands are creating a new era of increasing food prices.[19][20] Low-income urban populations are among the most vulnerable to food price increases, as they generally depend on the market for their food supplies and have limited budgets [21,22]. As prices rise, urban poor groups will be increasingly unable to afford a diet of the quality necessary to maintain their health [23]. There may also be other health consequences if the price increases drive them deeper into poverty, as some recent research suggests, [24] while riots and social instability can result from food shortages among low income urban populations.

Strategies that address the social determinants of health and health inequities promote adaptive capacity. They also correlate with sustainable development [25][26] and include improved infrastructure, education, institutional capacity and fairer access to resources, reduction of poverty and lessened intergenerational inequities. If the ultimate ambition of climate change adaptation is to (preferably) improve human well-being, which partly involves environmental protection, then it means that a broader approach that encompasses sustainability principles needs to be considered [27]. Pathways to adaptation are indeed part of sustainable development pathways [27], and a better understanding of the determinants of adaptive capacity will assist the building of these pathways. The linking or ‘mainstreaming’ of adaptation (and mitigation) activities into sustainable development policies is one way to more effectively respond to climate change [28-30]. In fact, it has been argued that sustainable development will necessarily take centre stage if we are to realign the demands of humans on our planet [31]. The current influx of funding for adaptation activities can be used strategically to unite these often disparate pathways of sustainable development and adaptation, of which health is a key component.

## Links between global health & climate change adaptation

The health field has attempted to align climate change adaptation to reduce health impacts in terms of the conventional public health categories of primary, secondary, and tertiary prevention and in some instances addresses the social determinants of health as described above [32-34]. Primary prevention in this context aims to intervene before disease or injury that may occur with climate change, such as rezoning coastal land to protect against rising sea levels and more intense extreme weather events such as coastal flooding. Primary prevention in this sense corresponds generally to anticipatory adaptation [35]. Secondary prevention involves the prevention of adverse health outcomes once disease has begun, but before it is symptomatic. Such approaches in the context of climate change include the strengthening of monitoring and surveillance of infectious diseases, and building public health and other infrastructure to better withstand extreme weather events and other likely incidents. Secondary prevention equates to reactive adaptation [35]. Tertiary prevention in public health aims to minimise the effects caused by existing disease. Tertiary prevention is the most reactive in this spectrum of adaptation and prevention actions, as the adverse health outcome is not prevented. An example of tertiary prevention is the improved treatment of manifest mental illness related to long-term drying. All of these prevention activities are beneficial and necessary for a healthy society; these measures constitute the basis of a “no-regrets” adaptation strategy [35]. This “no-regrets” approach to the health dimensions of climate change resonates with the fundamental humanitarian concerns underpinning much of the work on human vulnerability to ‘natural’ hazards and environmental change through its strong focus on minimise harm.

It could be argued that true primary prevention of the health effects of climate change involves averting climate change by the mitigation of greenhouse gas emissions, rather than responding to climate change events that are already occurring. The potential health co-benefits of mitigation activities have been well documented (*e.g.*[36,37]). Primary prevention could be described instead as “primary adaptive intervention”, as it is not genuine prevention. Adaptive strategies are perhaps better understood as secondary in nature as they recognise that climate change is occurring, and present options to reduce the potential health effects.

The World Health Organization’s (WHO) World Health Assembly of 2008 adopted a resolution urging Member States to take decisive action to address health impacts from climate change. An objective of this resolution was to strengthen health systems to respond to climate change impacts, which included the conduct of vulnerability and adaptation assessments and the prioritisation of further support [38]. Since then, regional arms of the WHO have been conducting vulnerability assessments to identify the health effects of climate change at country levels, and develop subsequent adaptation activities. The WHO has provided technical support for vulnerability and adaptation assessments in over 30 countries. For example, the Western Pacific Regional Office (WPRO) commissioned work in 2009 in Samoa, Cambodia and Mongolia with cooperation from country WHO offices. Prior to this, there had not been a clear and specific focus on public health, and inclusion of public health in NAPAs had generally been fairly weak. For example, the NAPA produced by Cambodia in 2006 only identified six direct public health projects out of a total of thirty-nine, and indeed, all of these were very narrow in their scope, only focusing on malaria. Bangladesh is another example where public health receives only a brief mention in its NAPA; a project on awareness-raising of the health effects of climate change, particularly focusing on communicable diseases, although health is mentioned as a sector for mainstreaming climate change adaptation activities and capacity building. A review of Annex 1 countries (industrialised countries and countries in transition) reported that no countries recognised health vulnerability within their Fifth National Communications [39].

## Current funding mechanisms for adaptation projects

The funding of adaptation activities to respond to unavoidable climate change has emerged as a major theme in climate change negotiations. There exist two maingroups–donors (generally developed countries) and recipients (developing countries). Again, equity is an important factor here–those most vulnerable to climate change are the ones that are least responsible for it, and this consequently underlines the responsibility of developed countries to provide substantial financial contributions. Donors’ motivation to fund adaptation activities is partly in response to UNFCCC negotiations which include the funding of adaptation activities; however it is also feasible that donors may understand and concur with the logical links between funding climate change adaptation and supporting broader development activities. This link, though, can also complicate the need to demonstrate additional funding for adaptation, if sustainable development activities are potentially rebadged as adaptation. Given the complicated nature of funding for adaptation, an improved understanding of governance structures and processes that influence the development and financing of adaptation activities, particularly in relation to health, is required [40].

A broad overview of the main climate change adaptation funding mechanisms has recently been conducted, in light of definitional issues relating to country eligibility for the current influx of adaptation financing measures [41]. The three mechanisms identified by this overview are the World Bank’s Pilot Program for Climate Resilience (PPCR), which sits within the Bank’s Climate Investment Fund established in 2008; the European Commission’s Global Climate Change Alliance of the European Union (GCCA), set up in 2007; and the Adaptation Fund (AF), operational since 2009 under the Kyoto Protocol to the UNFCCC. The different approaches used to identifying eligible countries for funding based on their levels of vulnerability has obvious implications for equitable resource allocation of adaptation funds. Although not included in Klein and Möhner’s review, the Global Environmental Fund (GEF), which was established in 1991 under the UNFCCC, is also a major funder of adaptation (and mitigation) activities and responsible for supporting NAPA processes in countries; in this sense, the GEF supports the processes that produce the necessary baseline data around vulnerability and potential adaptation activities The GEF and the AF, while closely connected (e.g. with the GEF providing the secretariat to the AF), are separate. 

All three funding bodies (PPCR, GCCA, AF) have identified the NAPA as a key basis for funding decisions in countries – this is either funding for implementing the NAPAs (*e.g.* PPCR), or funding for activities that are consistent with NAPAs (*e.g.* AF). It is unsurprising that the funding bodies link their programs with the NAPAs, however it is vital to bear in mind that the quality of these country-level assessments vary significantly, so although a consideration of their contents is important, it is most important to consider complementary country activities such as development plans, poverty reduction strategies, and in the case of public health, the consideration and where appropriate, the incorporation of health policies. Due to the NAPAs varying in their inclusion and analysis of the health effects of climate change, it may mean that the public health sector is not fully acknowledged when these funding bodies allocate adaptation resources. This is despite health being identified as one of the priority sectors identified by the NAPA guidelines. A recent WHO review found that less than one-quarter of the 41 NAPAs reviewed provided a comprehensive assessment of health vulnerability [42].

The Green Climate Fund (GCF), which sits within the UNFCCC, was agreed to in Cancun 2010 and is the latest in the iteration of climate change funding bodies. The purpose of this fund is to rapidly mobilise resources for adaptation and mitigation activities in developing countries. The stated objective of the GCF is to promote a paradigm shift towards low-emissions and climate-resilient development pathways. The fund has a financial goal of raising US$30billion by 2012 and US$100billion (from public and private sources) annually by 2020. An independent secretariat will support the fund’s operations. The GCF was approved at CoP17 in 2011, however there are still outstanding issues such as which country will host the Fund’s secretariat, selection of Board members and the nomination of a financial trustee. Financial arrangements will be finalised at CoP19. It would seem that it may become the most important international financial tool to respond to climate change, however the operation and funding structure of this fund is still unclear.

Currently, there is no specific focus on health within the funds described above, or the UNFCCC negotiation structure. Very little financial support is provided through the UNFCCC mechanisms to address the health impacts of climate change; less than 1% of the distribution of the Least Developed Countries Fund (LDCF), which finances the preparation and implementation of the NAPAs, has been allocated to health protection [43].

## Conclusion

Global health plays an integral role when it comes to developing policies and actions to adapt to climate change as it intersects many sectors that will be directly affected by climate change. The massive influx of new and additional funding that is labelled for climate change adaptation can be used strategically by the global health field as a lever to address the fundamental social causes of health and health inequities. Climate change will bring nothing new *per se* to our global health status; however our current burden of disease and social inequities will be exacerbated.

There are a number of well-financed climate change adaptation mechanisms that will continue to grow, along with the confusion that comes with many different players and poorly organised governance structures. Although the WHO has identified climate change as an issue to be addressed, funding for rigorous vulnerability assessments that focus on the health effects of climate change remains minimal. The potential over-reliance on the NAPAs as a key source for the prioritisation of adaptation funding is a concerning prospect, given the historical lack of focus on global health within these plans. Currently the WHO needs to compete with other players in the adaptation scene, many of which can produce quick and visible adaptation outcomes for monetary investment (such as flood walls, bridges), which is generally not the case for public health. In addition, the allocation of funding for adaptation is often dependent on competing local priorities (which may have unrecognised synergies with adaptation activities) and effective lobbying of and outreach to bilateral and multilateral donors.

In terms of future research, a review is required for Non-Annex 1 countries (developing countries) to clarify to what extent health is being considered within adaptation responses.

The global health community cannot see climate change adaptation as a threat to its other work; this is an opportunity to improve coherence and synergies across disciplines and sectors that contribute to human health.

## Competing interests

The authors declare that they have no competing interests.

## Authors’ contributions

KB & SF carried out the literature review and drafted the manuscript. Both authors read and approved the final manuscript. All authors read and approved the final manuscript.
